# Association between homocysteine levels and hyperlipidemia prevalence as well as all-cause mortality of hyperlipidemia patients in the US population: results from NHANES database

**DOI:** 10.3389/fcvm.2024.1419579

**Published:** 2024-07-25

**Authors:** Jia Wei, Qiang Yang, Xiaofang Wang, Xin He, Wenjing Zhu, Lin Lin, Chang Liu, Canzhan Zhu, Mingjuan Zhang

**Affiliations:** ^1^ Department of Cardiology, The Second Affiliated Hospital of Xi'an Jiaotong University, Xi'an, Shaanxi, China; ^2^ Department of Ultrasound, The Second Affiliated Hospital of Xi'an Jiaotong University, Xi'an, Shaanxi, China

**Keywords:** homocysteine, hyperlipidemia, NHANES, Kaplan-Meier estimate, cox proportional hazards analysis

## Abstract

**Objective:**

Several studies have investigated the correlation between blood lipids and homocysteine, but no clear conclusions have been defined yet. Therefore, we utilized data from National Health and Nutrition Examination Survey (NHANES) to explore the correlation between serum homocysteine (Hcy) levels and hyperlipidemia, which is determined by the levels of total cholesterol (TC), low-density lipoprotein cholesterol (LDL-C), high-density lipoprotein cholesterol (HDL-C), and triglycerides (TG). We believe this study can provide a scientific basis for the prevention and treatment of lipid abnormalities.

**Methods:**

The data used in this study were sourced from NHANES 1999–2006, linked with National Death Index mortality data from January 1999 to December 2019. We employed logistic regression to assess the associations between Hcy levels and the presence of hyperlipidemia. Additionally, survival analysis using Kaplan-Meier estimate and Cox proportional hazards regression model was conducted to evaluate the associations between Hcy levels and all-cause mortality in the hyperlipidemia population.

**Results:**

(1) A total of 13,661 subjects were included in the study. There were statistically significant differences in Hcy levels across different groups based on gender, age, race, marital status, education level, hypertension status, diabetes status, and Body Mass Index (BMI) (*P *< 0.05). (2) In the overall population, hyperhomocysteinemia (HHcy) was associated with an increased risk of high-TC hyperlipidemia (*P *< 0.05). Subgroup analysis by gender showed that HHcy in females was associated with an increased risk of dyslipidemia (OR = 1.30, 95% CI: 1.07–1.59, *P *< 0.05) and high-LDL-C hyperlipidemia (OR = 1.30, 95% CI: 1.00–1.68, *P *< 0.05). In addition, subgroup analysis by age revealed that HHcy in middle-aged people was associated with an increased risk of high-TC hyperlipidemia (OR = 1.21, 95% CI: 1.03–1.41, *P *< 0.05) and high-LDL-C hyperlipidemia (OR = 1.23, 95% CI: 1.06–1.43, *P *< 0.05). (3) HHcy was consistently associated with an increased mortality risk in the hyperlipidemia population (HR = 1.49, 95% CI: 1.35–1.65, *P *< 0.05).

**Conclusion:**

There was positive correlation between Hcy levels and the presence of hyperlipidemia. In the overall population, HHcy was associated with an increased risk of high-TC hyperlipidemia. Among females, HHcy is linked to an increased risk of dyslipidemia and high-LDL-C hyperlipidemia. In middle-aged people, HHcy was associated with an elevated risk of high-TC hyperlipidemia and high-LDL-C hyperlipidemia. In addition, HHcy increased the all-cause mortality rate in hyperlipidemia patients.

## Introduction

Hyperlipidemia is a common chronic noninfectious disease caused by abnormal lipoprotein metabolism in the human body. A large number of studies have shown that hyperlipidemia is an independent risk factor of cardiovascular and cerebrovascular diseases, including atherosclerosis, myocardial infarction, cerebral infarction and coronary heart disease (1). In the United States, approximately 28 million adults have a cholesterol level of 240 mg/dl or above, whose risk of developing Atherosclerotic Cardiovascular Disease (ASCVD) is about twice of those with normal lipid levels (2, 3). Hyperlipidemia has become a serious threat to human health. The research by Stevens W et al. (4) revealed that the recurrence and mortality of cardiovascular diseases showed a decreasing trend after effective measures were taken to control blood lipid levels. Therefore, there are significant clinical values to control blood lipid levels (5).

Homocysteine (Hcy) is a sulfur-containing amino acid, an intermediate product generated from the metabolism of methionine (6, 7). Several studies have demonstrated that the increase in serum Hcy levels is a strong risk factor of cardiovascular diseases (8, 9). According to a recent meta-analysis, the risk of cardiovascular diseases and stroke was elevated by 10% and 20%, respectively, for each 25% increase in plasma Hcy (10). Physiologically, Hcy may drive the process of cardiovascular diseases through various mechanisms including blood coagulant properties and oxidative stress-inducing injuries of vascular endothelium and arterial walls (11, 12).

A number of studies have explored the relationship between serum Hcy levels and hyperlipidemia. However, there have not been consistent conclusions (13). A study found that the C677T mutation in the methylenetetrahydrofolate reductase gene was closely related to hyperlipidemia, and this mutation could lead to Hcy metabolism disorders, resulting in hyperhomocysteinemia (HHcy) (14). Glueck et al. (15) analyzed 482 hyperlipidemia patients, 18 of whom were accompanied by HHcy. Among these 18 patients, 13 had atherosclerosis, with an incidence rate of 72%, much higher than that in hyperlipidemia patients with normal plasma Hcy levels.

The objective of this study is to examine the correlation between serum Hcy levels and the presence of hyperlipidemia in American adults. We believe that the findings from this research can provide valuable insights for the treatment and prevention of hyperlipidemia. The analysis utilized data from National Health and Nutrition Examination Survey (NHANES).

## Materials and methods

### Data source

NHANES is a nationwide initiative launched by the National Center for Health Statistics (NCHS) that primarily focuses on the health and nutritional conditions of U.S. civilians. The health data are collected and published at two-year intervals to obtain a comprehensive understanding of contemporary disease profiles and to inform public health policies (16–18). In this study, we used the data from NHANES 1999–2006 linked with National Death Index (NDI) mortality data collected from January 1999 to December 2019.

### Study population

**Figure 1:**
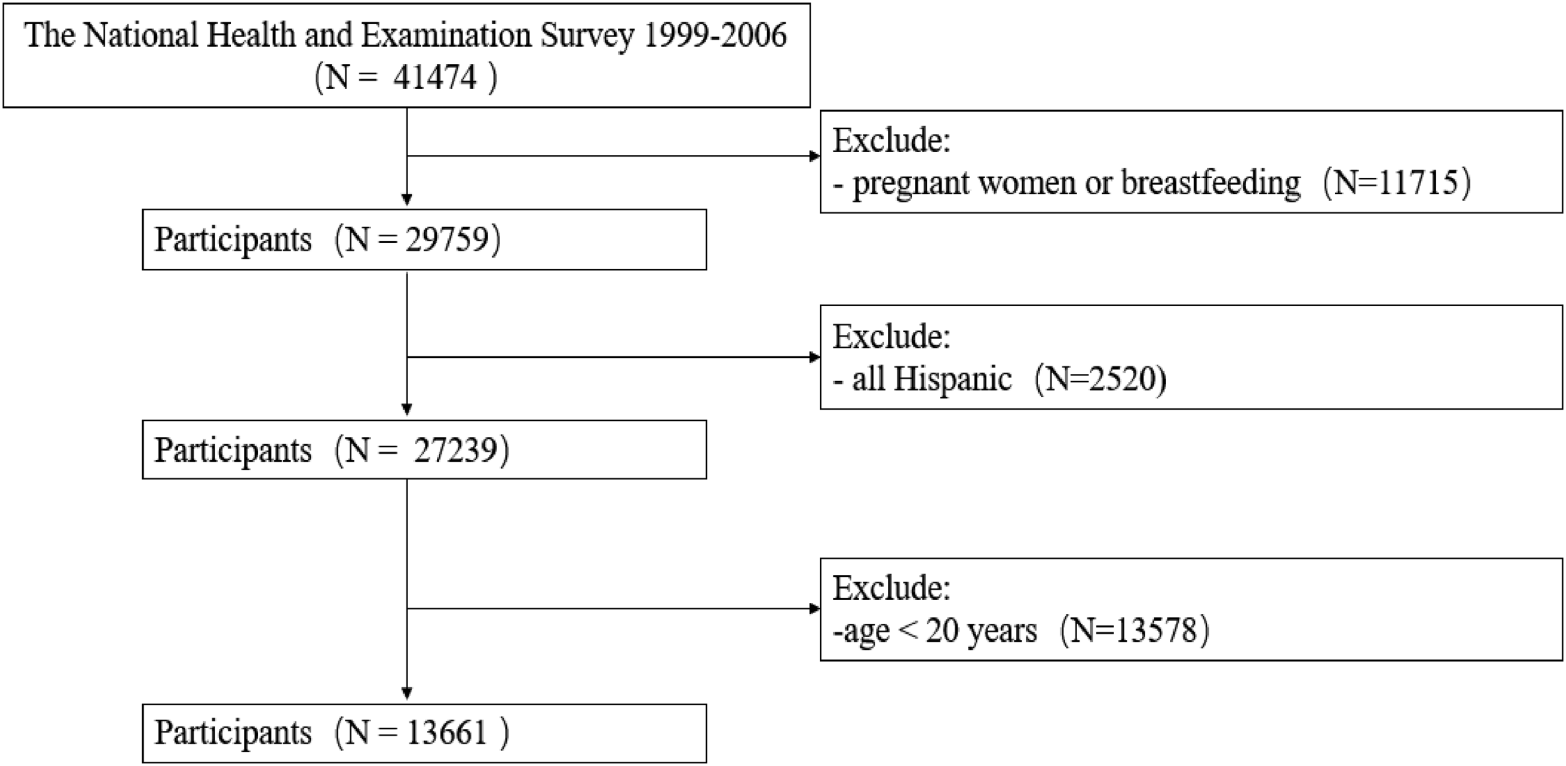
Flowchart depicting the participants selection.

We started with a total of 41,474 participants collected from NHANES 1999–2006. The pregnant or breastfeeding women were firstly excluded from the total samples. We also excluded Hispanic and non-Mexican-American Hispanic people following recommendations from NCHS (19). Additionally, participants younger than 20 years old were excluded. Eventually, a total of 13,661 subjects were included in the study. The participant selection criteria were illustrated in Figure 1.

### Discriminant criteria for hyperlipidemia and HHcy

Subjects were classified as having hyperlipidemia if their low-density lipoprotein (LDL), total cholesterol (TC), triglyceride (TG) or lipoprotein levels were greater than the 90th percentile compared to the general population, or if their high-density lipoprotein (HDL) levels were less than the 10th percentile. Specifically, high-TC hyperlipidemia was defined as TC ≥ 6.22 mmol/L (normal TC: 2.80–6.22 mmol/L). High-TG hyperlipidemia was defined as TG ≥ 2.26 mmol/L (normal TG: 0.45–2.25 mmol/L). High LDL-C hyperlipidemia was defined as LDL-C ≥ 4.14 mmol/L (normal LDL-C: 2.07–3.14 mmol/L). Low HDL-C hyperlipidemia was defined as HDL-C < 1.04 mmol/L (normal HDL-C: 1.04–1.45 mmol/L). Subjects with any one of the four types of hyperlipidemia were classified as having dyslipidemia.

HHcy was defined as the concentration of Hcy in the blood exceeding 15.4 μmol/L.

### Covariates

Gender, age, race/ethnicity, marital status, education level, Body Mass Index (BMI), hypertension status and diabetes status were included as covariates in the study. Age and BMI were continuous variables in the database and were converted to categorical variables, i.e., participants were categorized by age as youth (20–39 years old), middle-aged (40–59 years old) and elderly (60 years old and over), and were categorized by BMI as normal (18.5–24.9 kg/m^2^), overweight (18.5–24.9 kg/m^2^) and obesity (≥30 kg/m^2^). By Race/ethnicity, participants were classified as Mexican Americans, non-Hispanic white people and non-Hispanic black people. Marital status was divided into three groups: married, single and other. By education levels, participants were classified as below high school, high school graduate and above high school. Hypertension status was binary variables, i.e., participants were categorized as two groups: with hypertension and without hypertension. So was the diabetes status. Hypertension was determined by any one of the following criteria: (1) systolic blood pressure (SBP) ≥ 140 mm/Hg, (2) diastolic blood pressure (DBP) ≥ 90 mm/Hg, (3) subjects were told to had high blood pressure more than 2 times, (4) subjects were taking prescriptions for hypertension. Diabetes was determined by any one of the following criteria: (1) glucose ≥ 126 mg/dl, (2) glycohemoglobin ≥ 6.5%, (3) subjects were taking insulin or diabetic pills to lower blood sugar levels.

### Statistical analysis

Before statistical analysis, multiple imputation of missing samples for specific variables, including education level, marital status, Hcy, TC, TG, LDL, HDL, BMI, SBP, DBP, glucose and glycohemoglobin, were performed using the predictive mean matching approach. Continuous variables were then converted to categorical variables using the criteria described above, and were converted to counting variables subsequently. Group comparisons were conducted using the Rao-Scott-X^2^ test. Univariate and multivariate binary logistic regression analyses were performed to analyze the correlation between Hcy levels and hyperlipidemia, with adjustment for covariates in the multivariate models. In addition, subgroup analysis by gender and age were conducted to explore these associations within different gender and age groups.

All statistical analyses were performed with R software (version 4.3.2, https://www.R-project.org, the R Foundation). *P*-value < 0.05 was considered to be statistically significant.

## Results

### Data distribution before and after multiple imputation

**Figure 2:**
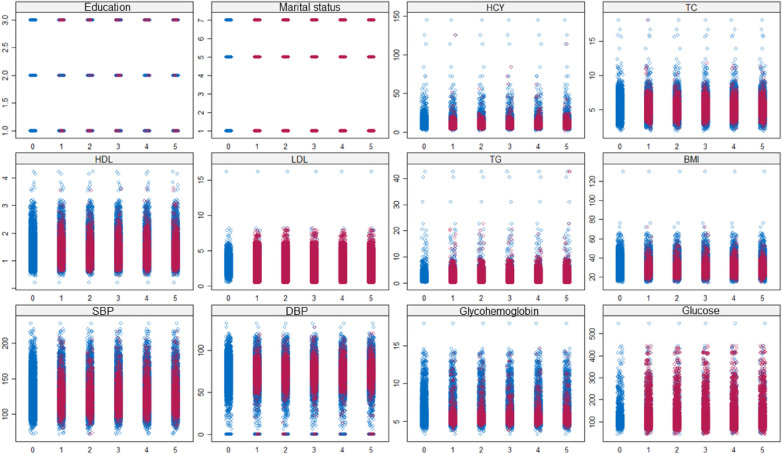
Data distribution before (blue plots) and after (red plots) multiple imputation. Coordinate 0 of horizontal axis indicated the raw data without imputation; coordinate 1-5 indicated the results of 5 imputations.

Figure 2 depicted the variable distribution before and after multiple imputation. The blue plots represented the data distribution without imputation, while the red plots indicated the data distribution after imputation. The coordinate 0 of the horizontal axis showed the raw data before imputation, and the coordinates 1–5 indicated the results after 5 cycles of imputation. The figure illustrated that the interpolated values closely approximated the distribution of the raw data, indicating that the multiple imputation was able to effectively handle the missing values, and thereby could increase the reliability of statistical analysis.

### Baseline characteristics

The baseline characteristics of the participants categorized by Hcy levels were presented in Table 1. The analysis enrolled a total of 13,661 eligible participants, of whom 65% were men and 35% were women. The results showed that there were statistically significant differences in the distribution of Hcy levels across different sex, age, race, marital status, education level, BMI, hypertension and diabetes groups (*P *<* *0.05). Compared with the participants having normal Hcy levels, those with HHcy tended to be male, elderly, non-Hispanic white people, with education of less than high school, have hypertension and have diabetes.

**Table 1:** Comparison of characteristics according to the Hcy levels (*n* = 13,661).

Characteristic	*n* (%)	Normal Hcy	HHcy	*χ* ^2^	*P*
Total	13,661 (100)	10,210 (74.7)	3,451 (25.3)		
Gender				974.53	<0.01
Male	8,885 (65.0)	5,884 (57.6)	3,001 (87.0)		
Female	4,776 (35.0)	4,326 (42.4)	450 (13.0)		
Age				1,929.4	<0.01
Youth	5,225 (38.2)	4,626 (45.3)	599 (17.4)		
Middle	5,108 (37.4)	4,021 (39.4)	1,087 (31.5)		
Elderly	3,328 (24.4)	1,563 (15.3)	1,765 (51.1)		
Race/ethnicity				140.01	<0.01
Mexican American	3,253 (23.8)	2,676 (26.2)	577 (16.7)		
Non-Hispanic white	7,300 (53.4)	5,214 (51.1)	2,086 (60.4)		
Non-Hispanic black	3,108 (22.8)	2,320 (22.7)	788 (22.8)		
Education				76.837	<0.01
Less than high school	4,081 (29.9)	2,869 (28.1)	1,212 (35.1)		
High school	3,227 (23.6)	2,392 (23.4)	835 (24.2)		
Above high school	6,353 (46.5)	4,949 (48.5)	1,404 (40.7)		
Marital status				108.26	<0.01
Married	7,913 (57.9)	5,838 (57.2)	2,075 (60.1)		
Single	2,496 (18.3)	2,061 (20.2)	435 (12.6)		
Other	3,252 (23.8)	2,311 (22.6)	941 (27.3)		
BMI				14.137	0.01
Normal	4,217 (30.9)	3,210 (31.4)	1,007 (29.2)		
Overweight	4,917 (36.0)	3,585 (35.1)	1,332 (38.6)		
Obesity	4,527 (33.1)	3,415 (33.4)	1,112 (32.2)		
Hypertension				762.79	<0.01
No	8,930 (65.4)	7,342 (71.9)	1,588 (46.0)		
Yes	4,731 (34.6)	2,868 (28.1)	1,863 (54.0)		
Diabetes				172.97	<0.01
No	11,998 (87.8)	9,186 (90.0)	2,812 (81.5)		
Yes	1,663 (12.2)	1,024 (10.0)	639 (18.5)		

Baseline characteristics according to the presence of dyslipidemia, high-TC hyperlipidemia, high-TG hyperlipidemia, low-HDL-C hyperlipidemia and high-LDL-C hyperlipidemia were shown in Table 2. The results showed that there were statistically significant differences in the presence of dyslipidemia among different gender, age, race, marital status, education level, hypertension, diabetes and BMI groups (*P *< 0.05).

**Table 2:** Comparison of characteristics according to the presence of dyslipidemia, high-TC hyperlipidemia, high-TG hyperlipidemia, low-HDL-C hyperlipidemia and high-LDL-C hyperlipidemia.

Characteristic	Dyslipidemia		*P*	High-TC hyperlipidemia		*P*	High-TG hyperlipidemia		*P*	Low-HDL-C hyperlipidemia		*P*	Low-HDL-C hyperlipidemia		*P*
	No	Yes		No	Yes		No	Yes		No	Yes		No	Yes	
Total	6,832	6,829		11,804	1,857		10,780	2,881		10,199	3,462		11,450	2,211	
Gender			<0.01			<0.01			<0.01			<0.01			<0.01
Male	4,029 (59.0)	4,856 (71.1)		7,625 (64.6)	1,260 (67.9)		6,949 (64.5)	1,936 (67.2)		6,106 (59.9)	2,779 (80.3)		7,302 (63.8)	1,583 (71.6)	
Female	2,803 (41.0)	1,973 (28.9)		4,179 (35.4)	597 (32.1)		3,831 (35.5)	945 (32.8)		4,093 (40.1)	683 (19.7)		4,148 (36.2)	628 (28.4)	
Age			<0.01			<0.01			<0.01			<0.01			<0.01
Youth	2,850 (41.7)	2,375 (34.8)		4,737 (40.1)	488 (26.3)		4,214 (39.1)	1,011 (35.1)		3,954 (38.8)	1,271 (36.7)		4,580 (40.0)	645 (29.2)	
Middle	2,752 (40.3)	3,107 (45.5)		4,798 (40.6)	1,061 (57.1)		4,556 (42.3)	1,303 (45.2)		4,427 (43.4)	1,432 (41.4)		4,658 (40.7)	1,201 (54.3)	
Elderly	1,230 (18.0)	1,347 (19.7)		2,269 (19.2)	308 (16.6)		2,010 (18.6)	567 (19.7)		1,818 (17.8)	759 (21.9)		2,212 (19.3)	365 (16.5)	
Race/ethnicity			<0.01			<0.01			<0.01			<0.01			<0.01
Mexican American	1,517 (22.2)	1,736 (25.4)		2,801 (23.7)	452 (24.3)		2,522 (23.4)	731 (25.4)		2,314 (22.7)	939 (27.1)		2,685 (23.4)	568 (25.7)	
Non-Hispanic white	3,563 (52.2)	3,737 (54.7)		6,248 (52.9)	1,052 (56.7)		5,707 (52.9)	1,593 (55.3)		5,382 (52.8)	1,918 (55.4)		6,091 (53.2)	1,209 (54.7)	
Non-Hispanic black	1,752 (25.6)	1,356 (19.9)		2,755 (23.3)	353 (19.0)		2,551 (23.7)	557 (19.3)		2,503 (24.5)	605 (17.5)		2,674 (23.4)	434 (19.6)	
Education			<0.01			<0.01			<0.01			<0.01			<0.01
<high school	1,908 (27.9)	2,176 (31.9)		3,509 (29.7)	575 (31.0)		3,224 (29.9)	860 (29.9)		2,876 (28.2)	1,208 (34.9)		3,387 (29.6)	697 (31.5)	
High school	1,516 (22.2)	1,714 (25.1)		2,749 (23.3)	481 (25.9)		2,488 (23.1)	742 (25.8)		2,329 (22.8)	901 (26.0)		2,652 (23.2)	578 (26.1)	
>High school	3,408 (49.9)	2,939 (43.0)		5,546 (47.0)	801 (43.1)		5,068 (47.0)	1,279 (44.4)		4,994 (49.0)	1,353 (39.1)		5,411 (47.3)	936 (42.3)	
Marital status			<0.01			<0.01			0.44			<0.01			<0.01
Married	3,837 (56.2)	4,073 (59.6)		6,761 (57.3)	1,149 (61.9)		6,227 (57.8)	1,683 (58.4)		5,813 (57.0)	2,097 (60.6)		6,531 (57.0)	1,379 (62.4)	
Single	1,376 (20.1)	1,102 (16.1)		2,231 (18.9)	247 (13.3)		1,979 (18.4)	499 (17.3)		1,935 (19.0)	543 (15.7)		2,175 (19.0)	303 (13.7)	
Other	1,619 (23.7)	1,654 (24.2)		2,812 (23.8)	461 (24.8)		2,574 (23.9)	699 (24.3)		2,451 (24.0)	822 (23.7)		2,744 (24.0)	529 (23.9)	
BMI			<0.01			<0.01			<0.01			<0.01			<0.01
Normal	2,618 (38.3)	1,569 (23.0)		3,779 (32.0)	408 (22.0)		3,410 (31.6)	777 (27.0)		3,637 (35.7)	550 (15.9)		3,769 (32.9)	418 (18.9)	
Overweight	2,352 (34.4)	2,589 (37.9)		4,171 (35.3)	770 (41.5)		3,861 (35.8)	1,080 (37.5)		3,625 (35.5)	1,316 (38.0)		4,038 (35.3)	903 (40.8)	
Obesity	1,862 (27.3)	2,671 (39.1)		3,854 (32.6)	679 (36.6)		3,509 (32.6)	1,024 (35.5)		2,937 (28.8)	1,596 (46.1)		3,643 (31.8)	890 (40.3)	
Hypertension			<0.01			<0.01			<0.01			<0.01			<0.01
No	4,722 (69.1)	4,245 (62.2)		7,870 (66.7)	1,097 (59.1)		7,167 (66.5)	1,800 (62.5)		6,858 (67.2)	2,109 (60.9)		7,617 (66.5)	1,350 (61.1)	
Yes	2,110 (30.9)	2,584 (37.8)		3,934 (33.3)	760 (40.9)		3,613 (33.5)	1,081 (37.5)		3,341 (32.8)	1,353 (39.1)		3,833 (33.5)	861 (38.9)	
Diabetes			<0.01			0.19			<0.01			<0.01			0.56
No	6,197 (90.7)	5,799 (84.9)		10,383 (88.0)	1,613 (86.9)		9,614 (89.2)	2,382 (82.7)		9,161 (89.8)	2,835 (81.9)		10,063 (87.9)	1,933 (87.4)	
Yes	635 (9.3)	1,030 (15.1)		1,421 (12.0)	244 (13.1)		1,166 (10.8)	499 (17.3)		1,038 (10.2)	627 (18.1)		1,387 (12.1)	278 (12.6)	

### Association of Hcy levels with the presence of dyslipidemia

As shown in Table 3, we performed binary logistic regression analysis to identify the association of Hcy levels with the presence of dyslipidemia. The results from the univariate model indicated that HHcy was associated with an increased risk of dyslipidemia (OR = 1.21, 95% CI: 1.11–1.30, *P *< 0.01), high-TC hyperlipidemia (OR = 1.21, 95% CI: 1.08–1.35, *P *< 0.01), high-LDL-C hyperlipidemia (OR = 1.17, 95% CI: 1.05–1.29, *P *< 0.01) and low-HDL-C hyperlipidemia (OR = 1.25, 95% CI: 1.15–1.37, *P *< 0.01). However, after adjusting for potential confounders, the associations of HHcy with dyslipidemia, low-HDL-C hyperlipidemia or high-LDL-C hyperlipidemia changed significantly (*P > *0.05), which implied that the covariates had a significant impact to these associations.

**Table 3:** Logistic regression analysis on the association of HHcy with the presence of dyslipidemia.

	HHcy		*P*
	No	Yes	
Dyslipidemia			
Model 1: OR [95% CI]	1.00 (ref.)	1.21 [1.11–1.30]	<0.01
Model 2: OR [95% CI]	1.00 (ref.)	1.04 [0.95–1.13]	0.36
Model 3: OR [95% CI]	1.00 (ref.)	1.02 [0.94–1.11]	0.62
High-TC hyperlipidemia			
Model 1: OR [95% CI]	1.00 (ref.)	1.21 [1.08–1.35]	<0.01
Model 2: OR [95% CI]	1.00 (ref.)	1.16 [1.03 1.31]	<0.05
Model 3: OR [95% CI]	1.00 (ref.)	1.13 [1.00–1.27]	<0.05
High-TG hyperlipidemia			
Model 1: OR [95% CI]	1.00 (ref.)	1.07 [0.97 1.18]	0.16
Model 2: OR [95% CI]	1.00 (ref.)	1.02 [0.92–1.13]	0.66
Model 3: OR [95% CI]	1.00 (ref.)	1.02 [0.91–1.13]	0.43
High-LDL-C hyperlipidemia			
Model 1: OR [95% CI]	1.00 (ref.)	1.17 [1.05–1.29]	<0.01
Model 2: OR [95% CI]	1.00 (ref.)	1.06 [0.95–1.19]	0.27
Model 3: OR [95% CI]	1.00 (ref.)	1.05 [0.93–1.17]	0.43
Low-HDL-C hyperlipidemia			
Model 1: OR [95% CI]	1.00 (ref.)	1.25 [1.15–1.37]	<0.01
Model 2: OR [95% CI]	1.00 (ref.)	1.01 [0.92–1.11]	0.79
Model 3: OR [95% CI]	1.00 (ref.)	1.00 [0.90–1.10]	0.95

Model 1: univariate model.

Model 2: adjusted for gender, age, and race.

Model 3: further adjusted for marital status, education level, hypertension status, diabetes status, and BMI on the basis of model 2.

### Subgroup analysis

In order to explore and characterize the influence of covariates, we performed subgroup multivariate logistic analysis by gender and age, and the results were presented in Table 4. The findings showed that HHcy was associated with an increased risk of dyslipidemia (OR = 1.30, 95% CI: 1.07–1.59, *P *< 0.05), and high-LDL-C hyperlipidemia (OR = 1.30, 95% CI: 1.00–1.68, *P *< 0.05) in female. However, in male, there was no statistically significant association (*P *> 0.05) observed. It was noteworthy that some OR values were less than 1, suggesting that HHcy might potentially act as a protective factor for dyslipidemia, high-TG hyperlipidemia and low-HDL-C hyperlipidemia. However, these findings were not conclusive and further investigations were needed.

**Table 4:** Association of HHcy with dyslipidemia according to gender and age.

	HHcy		*P*
	No	Yes	
Dyslipidemia			
By gender^a^: OR [95% CI]			
Male	1.00 (ref.)	0.97 [0.88 1.07]	0.56
Female	1.00 (ref.)	1.30 [1.07–1.59]	<0.01
By age^b^: OR [95% CI]			
Youth	1.00 (ref.)	1.03 [0.86–1.23]	0.74
Middle	1.00 (ref.)	1.09 [0.94–1.25]	0.25
Elderly	1.00 (ref.)	0.95 [0.83–1.10]	0.51
High-TC hyperlipidemia			
By gender: OR [95% CI]			
Male	1.00 (ref.)	1.11 [0.97–1.27]	0.12
Female	1.00 (ref.)	1.22 [0.95–1.55]	0.10
By age: OR [95% CI]			
Youth	1.00 (ref.)	1.13 [0.84–1.49]	0.40
Middle	1.00 (ref.)	1.21 [1.03–1.41]	<0.05
Elderly	1.00 (ref.)	0.99 [0.80–1.21]	0.91
High-TG hyperlipidemia			
By gender: OR [95% CI]			
Male	1.00 (ref.)	0.97 [0.87–1.09]	0.66
Female	1.00 (ref.)	1.22 [0.95–1.55]	0.11
By age: OR [95% CI]			
Youth	1.00 (ref.)	1.16 [0.93–1.44]	0.18
Middle	1.00 (ref.)	0.98 [0.83–1.16]	0.82
Elderly	1.00 (ref.)	0.98 [0.82–1.16]	0.81
High-LDL-C hyperlipidemia			
By gender: OR [95% CI]			
Male	1.00 (ref.)	1.01 [0.89–1.14]	0.88
Female	1.00 (ref.)	1.30 [1.00–1.68]	<0.05
By age: OR [95% CI]			
Youth	1.00 (ref.)	1.16 [0.90–1.48]	0.24
Middle	1.00 (ref.)	1.23 [1.06–1.43]	<0.01
Elderly	1.00 (ref.)	0.92 [0.73–1.16]	0.48
Low-HDL-C hyperlipidemia			
By gender: OR [95% CI]			
Male	1.00 (ref.)	0.96 [0.86–1.07]	0.51
Female	1.00 (ref.)	1.25 [0.93–1.64]	0.13
By age: OR [95% CI]			
Youth	1.00 (ref.)	0.81 [0.65–1.00]	0.06
Middle	1.00 (ref.)	0.92 [0.77–1.08]	0.33
Elderly	1.00 (ref.)	1.12 [0.94–1.34]	0.20

^a^ model is adjusted for age, race, education, marital status, BMI, hypertension status and diabetes status.

^b^ model is adjusted for gender, race, education, marital status, BMI, hypertension status and diabetes status.

Subgroup analysis by age revealed that HHcy in middle-aged individuals was associated with an increased risk of high-TC hyperlipidemia (OR = 1.21, 95% CI: 1.03–1.41, *P *<* *0.05) and High-LDL-C hyperlipidemia (OR = 1.23, 95% CI: 1.06–1.43, *P *<* *0.05). However, in young and elderly people, the associations were not statistically significant (*P *> 0.05).

### Survival analysis

**Figure 3:**
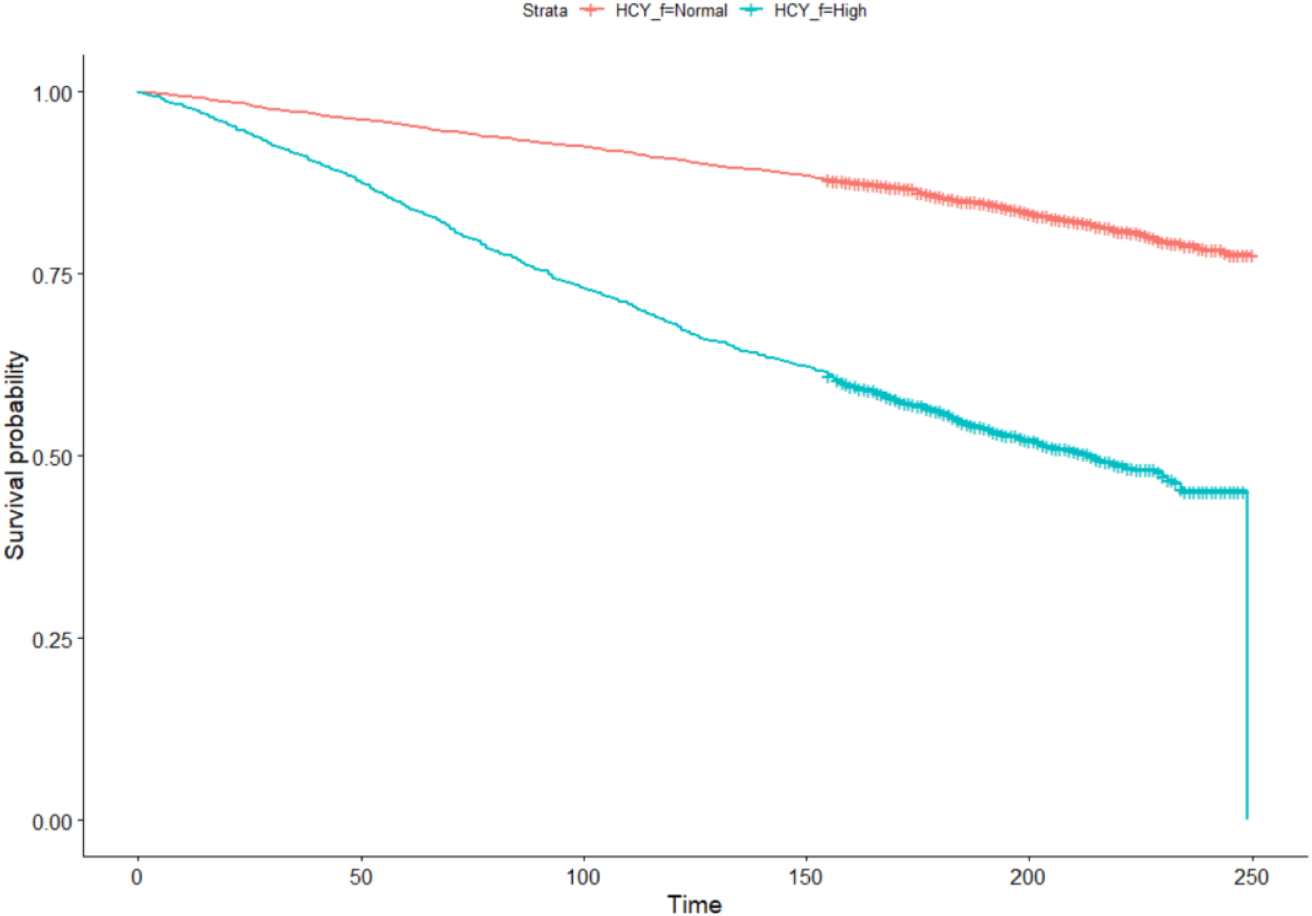
Univariate Kaplan-Meier curves. The red plot indicated the survival curve of subjects with normal Hcy, while the blue plot indicated the survival curve of subjects with HHcy.

Univariate Kaplan-Meier curves (Figure 3) suggested that HHcy is associated with a reduced survival probability in hyperlipidemia patients (*P *<* *0.05).

**Figure 4:**
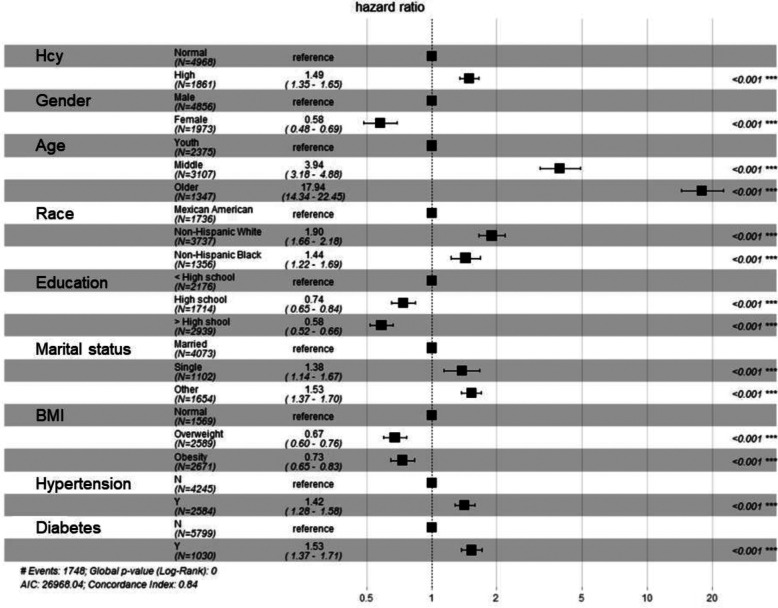
Forest plot for multivariate Cox regression analysis.

Multivariate Cox regression analysis after adjusting for sex, age, race, education, marriage, BMI, hypertension, and diabetes showed that HHcy was consistently associated with an increased mortality risk (HR = 1.49, 95% CI: 1.35–1.65) (Figure 4).

## Discussion

In this study, we explored the association between Hcy levels and the risk of dyslipidemia in the U.S. adults. After adjusting for covariates, HHcy was found to be closely associated with an increased risk of dyslipidemia and increased mortality risk in the dyslipidemia population. The associations remained significant in the female and middle-aged participants.

Cholesterol is a major component of cellular membranes. Hypercholesterolemia is the presence of a high amount of lipids in the blood (20). The association between dyslipidemia and Hcy had been examined in several previous studies (13, 21, 22), but the relationship between HHcy and hyperlipidemia have not been conclusively proved until now (23). Nabipour et al. (24) found significant associations between lower HDL cholesterol and high Hcy levels.

Single factor analysis showed that HHcy was associated with an increased risk of dyslipidemia, high-TC hyperlipidemia, low-HDL-C hyperlipidemia and high-LDL-C hyperlipidemia, whereas the association with high-TG hyperlipidemia was not significant. However, after adjusting for covariates, significant changes were observed in the associations of HHcy with dyslipidemia, low-HDL-C hyperlipidemia and high-LDL-C hyperlipidemia. Specifically, after adjusting solely for gender, the associations were no longer statistically significant, suggesting that the mechanism of HHcy interacting with hyperlipidemia might be different in male and female. Subgroup analysis by gender revealed notable differences in the impact of HHcy on dyslipidemia between men and women. Specifically, the associations between HHcy and the presence of dyslipidemia and high-LDL-C hyperlipidemia were more significant in women, while these associations were weaker in men. This may be caused by variations in metabolism, hormone levels and lifestyle between men and women. This discovery provided a new perspective on the prevention and treatment of cardiovascular diseases in different genders. For women with HHcy, special attention should be paid to the management of dyslipidemia to reduce the risk of mortality. Moreover, it is crucial to develop personalized treatment plans for patients of different genders.

It was observed that HHcy was closed associated with an increased risk of high-TC hyperlipidemia in the overall population, but the association was not statistically significant when analyzed separately in male and female subgroups. This phenomenon is known as Simpson's Paradox (25, 26). Further investigations are necessary to provide a reasonable interpretation of this finding.

Subgroup analysis by age showed that HHcy in middle-aged people was associated with an increased risk of high-TC hyperlipidemia and high-LDL hyperlipidemia. However, no significant correlation was found in young and elderly people. Previous studies have identified age as a significant risk factor for increased cardiovascular mortality due to HHcy (27). As age increases, the metabolism and physiological functions in the human body will change significantly, which may influence the correlation between HHcy and dyslipidemia. In young and elderly individuals, due to the combined effects of various factors including hormonal levels, lifestyle and genetic factors, etc., the direct association between HHcy and dyslipidemia may become less dominant. In summary, Hcy serves as an independent pathogenic factor of cardiovascular disease and demonstrates a certain correlation with hyperlipidemia. Elevated levels of Hcy or abnormal blood lipid can contribute to cardiovascular diseases and should be given sufficient attention in clinical management.

The strengths of this study lie in application of multiple imputation for missing data and adjustment for covariates including age, gender, and race etc. There are some limitations in this study. First, the ethnicity of the study subjects did not cover the whole American population. In the study, three racial groups were involved: Mexican American people, non-Hispanic white people and non-Hispanic black people. Other Hispanic individuals and other races were excluded based on recommendations from NCHS, as the sample size of these racial groups in the NHANES 1999–2006 dataset was too small to produce reliable estimates. Although NHANES oversampled the Hispanic subgroup in the 2007–2010 survey periods, the Hcy data were not included and published in these periods. Consequently, the findings of this study may not be broadly applicable to Hispanic people and other racial groups. In the future, including samples from these racial groups would be helpful to enhance the generalizability of the study conclusions. Second, this research cannot infer the causal relationship between HHcy and dyslipidemia. Prospective studies with larger samples are needed to further explore the causal relationship between the two variables. Third, this study only involved data samples of 1999–2006 survey periods because Hcy data were not published in the database after 2006. This significantly limited the sample size used in the analysis. Despite efforts like multiple imputation to interpolate the missing data and improve the reliability of estimates, gaps in data collection could introduce bias or reduce the accuracy of predictive models for future health outcomes. Furthermore, data from these periods may not fully capture changes in demographic shifts or emerging health trends over subsequent years, and may not fully reflect current health behaviors. With the changes in lifestyle, metabolic levels and healthcare practices, the correlation between HHcy and hyperlipidemia may undergo slight variations. Despite the above limitations, the obtained sample provides valuable insights into historical health trends and a foundational understanding of risk factors. It also serves as a benchmark for evaluating changes in health indicators. Researchers can leverage the findings of this study to forecast potential health outcomes and formulate health policies for the prevention of lipid abnormalities.

## Conclusion

HHcy is positively associated with hyperlipidemia. In the whole population, HHcy is associated with an increased risk of high-TC hyperlipidemia. In female, HHcy is associated with an increased risk of dyslipidemia and high-LDL-C hyperlipidemia. In middle-aged people, HHcy is associated with an increased risk of high-TC hyperlipidemia and high-LDL-C hyperlipidemia. HHcy can significantly increase the mortality rate of hyperlipidemia patients.

## Data Availability

The raw data supporting the conclusions of this article will be made available by the authors, without undue reservation.
